# Changes in Meat/Poultry/Fish Consumption in Australia: From 1995 to 2011–2012

**DOI:** 10.3390/nu8120753

**Published:** 2016-11-24

**Authors:** Zhixian Sui, David Raubenheimer, Judy Cunningham, Anna Rangan

**Affiliations:** 1 Charles Perkins Centre, School of Life and Environmental Sciences, The University of Sydney, Sydney, NSW 2006, Australia; david.raubenheimer@sydney.edu.au (D.R.); anna.rangan@sydney.edu.au (A.R.); 2 42 Caroline Street, Annerley, OLD 4103, Australia; judyc121@gmail.com

**Keywords:** red meat, poultry, processed meat, fish/seafood, dietary pattern

## Abstract

The purpose of the study was to examine temporal changes in meat/poultry/fish consumption patterns between 1995 and 2011–2012 in the Australian population. Meat/poultry/fish consumption from all food sources, including recipes, was analysed by gender, age group, and socio-economic status using 24-h recall data from the 1995 National Nutrition Survey (*n* = 13,858) and the 2011–2012 National Nutrition and Physical Activity Survey (*n* = 12,153). The overall proportion of people consuming meat/poultry/fish remained stable (91.7% versus 91.3%, *p* = 0.55), but a shift in the type of meat consumed was observed. Red meat, including beef and lamb, was consumed by fewer people over the time period (from 56% to 49%), whereas poultry consumption increased (from 29% to 38%). Amounts of all meat/poultry/fish consumed were reportedly higher in 2011–2012 compared with 1995. This resulted in similar (red meat, and processed meat) or slightly higher (poultry, and fish) per-capita intakes in 2011–2012. The magnitude of change of consumption varied between children and adults, and by gender. Monitoring trends in consumption is particularly relevant to policy makers, researchers and other health professionals for the formulation of dietary recommendations and estimation of potential health outcomes.

## 1. Introduction

Meat, poultry and fish are an integral part of the Australian diet and provide essential nutrients such as protein, iron, zinc, vitamin B12 and essential fatty acids [1]. Recent apparent consumption and market data from Australia, and other countries such as the USA [2,3,4,5,6] suggest there has been an overall increase in the intake of meat/poultry/fish from the 1990s to 2011–2012, driven by a rise in the consumption of poultry and fish/seafood, and a simultaneous small decline in red meat. While such data are useful for capturing trends, they are not an accurate measure of meat consumption, as they do not account for waste, non-human consumption and the impact of global meat supplies. No detailed meat consumption data are currently available to examine changes over time using national dietary surveys. 

Two large nationally representative nutrition surveys have been conducted in Australia. The 1995 National Nutrition Survey (1995 NNS) [7] reported that on average Australian adults consumed 183.1 g of meat/poultry/fish per person on the day surveyed. The 2011–2012 National Nutrition and Physical Activity Survey (2011–2012 NNPAS), using an updated database of food items, reported that on average Australian adults consumed 182.9 g of meat/poultry/fish on the day surveyed [8]. However, these reports were based on broad food group analysis using meat/poultry/fish categories such as “beef and dishes” or “meat, meat dishes and meat products”. These food categories included the weight of ingredients other than meat/poultry/fish in meat-based dishes (for example the vegetables in a lamb stew), and excluded meat components of cereal-based dishes where meat was a minor ingredient (for example ham on pizza, or beef in meat pie). In addition, the coding of mixed dishes differed between the two surveys disabling a direct comparison of meat consumption. However, these challenges were resolved in the present analysis by disaggregating the meat components from all food components and mixed dishes in a similar manner for both surveys.

This paper uses disaggregated meat/poultry/fish consumption data using the 1995 NNS and 2011–2012 NNPAS. The aim was to provide a comprehensive overview of changes in meat/poultry/fish consumption over this 16-year period, taking gender, age, and socio-economic factors into consideration. These findings will help to inform evidence-based dietary advice and assist in the development and monitoring of nutrition and food policies and public health messages. 

## 2. Materials and Methods

### 2.1. The Surveys

The 1995 NNS collected information on food and nutrition from 3007 children and 10,851 adults by the Australian Bureau of Statistics (ABS) and the Commonwealth Department of Health and Family Services (HFS). The survey was conducted between February 1995 and March 1996 and included people from very remote areas. The 1995 NNS used one 24-h recall to collect food and beverage intake and a second 24-h recall was collected in a subset of 10% of the respondents for nutrient intake adjustment purposes.

The 2011–2012 NNPAS was undertaken by the ABS between May 2011 and June 2012. The survey contains 2812 children and 9341 adults in Australia but excluded people from very remote areas. All respondents were interviewed face-to-face for the collection of dietary intake data using a 24-h recall and a second recall was collected from 7735 (64%) respondents via a telephone interview. Further details about the scope and the methodology of the surveys are available from the ABS [7,8].

The characteristics of both surveys are similar (Supplementary Materials Table S1). The 2011–2012 NNPAS was designed to facilitate comparisons to the 1995 NNS where possible. Ethics approval for both surveys was granted by the Australian Government Department of Health and Ageing Departmental Ethics Committee. Purpose designed food composition databases were developed for each of the surveys (AUSNUT 1999 and AUSNUT 2011–2013). To take account of possible seasonal effects on health and nutrition characteristics, both surveys were spread across an approximately 12-month enumeration period. Only the first 24-h recall from each survey was analysed to enable equal comparison between the two time points as: (a) many types of meat/poultry/fish were consumed episodically and “usual intakes” could not be estimated; and (b) to enable direct comparison of proportions consuming food types [7,8].

### 2.2. Meat/Poultry/Fish Disaggregation

To assess overall meat/poultry/fish consumption, intake from all sources (individually recorded items and mixed dishes with meat/poultry/fish as a major or minor component) was estimated. The meat/poultry/fish components from mixed dishes were calculated using the AUSNUT 2011–2013 recipe file [9]. For example, the exact meat component (in grams) of a spaghetti bolognese, fried chicken, or a stir fry was extracted using the AUSNUT 2011–2013 recipe file [9] if individual ingredients were not recorded by the survey participant. The 2011–2012 NNPAS contained more foods than the 1995 NNS (5740 vs. 4550) largely due to the increased number of mixed dishes reported (1545 vs. 547). The linkage file provided by Food Standards Australia and New Zealand (FSANZ) [10] was used to match food types between the 1995 NNS and 2011–2012 NNPAS. The quantities of each individual meat type were aggregated to obtain an estimate of consumption per individual per day.

### 2.3. Categorisation of Meats

The term “meat/poultry/fish” as used in this study excludes eggs, tofu, nuts and seeds, legumes and beans. “Red meat” refers to beef (including veal), lamb (including mutton), pork, kangaroo, and game meats (including goat, venison, and rabbit) [1]. It includes muscle meat only, not offal. “Poultry” refers to chicken, duck and other poultry. “Fish/seafood” refers to all fresh finfish, seafood, canned fish, and fish/seafood products. All organ and offal meats were reported together because of the low frequency of consumption on a population level. “Processed meat” includes sausages, bacon, ham, nuggets, salami and other fermented meats. A detailed categorization of meat/poultry/fish can be found in Supplementary Materials Table S2. 

### 2.4. Statistical Analysis

Statistical analyses were performed using SPSS for Windows 22.0 software (SPSS Inc., Chicago, IL, USA). Per capita information represents the average intake of meat/poultry/fish over the whole population, whereas per-consumer information represents the average intake of every type of meat/poultry/fish only by those who reported consuming this food type in their 24 h recall. Descriptive statistics were used to report the proportion of consumers and per-capita consumption (average intake among the whole population) according to gender and age group. Socio-economic status was ranked based on quintiles of the Socio-Economic Index of Disadvantage for Areas (SEIFA), where the first SEIFA quintile indicates the most disadvantaged areas. SEIFA quintile rankings for all participants were supplied by the ABS, combining measures deemed to represent different aspects of relative socio-economic conditions in an area. Median intakes with the 25th and 75th quartile were also reported for per-consumer information (average intake among the consumers of individual types of meat/poultry/fish). Analysis of variance and Chi square tests were performed where appropriate to test the relationship between the proportion of intake and age, gender, or SEIFA categories. Differences in consumption between the two surveys were analysed using chi square, independent t-tests and Mann–Whitney U tests for non-parametric distributions. For all tests, a *p*-value of <0.05 was considered statistically significant. 

## 3. Results

### 3.1. Proportion

The total proportion of people who reported consuming meat/poultry/fish remained unchanged from 1995 to 2011 (91.7% versus 91.3%) although the proportions consuming various categories of meat/poultry/fish have changed (Table 1). Specifically, the proportion of people consuming red meat on the day of the survey declined from 56.1% to 48.6% (or 13% decrease), and processed meat decreased from 44.0% to 37.8% (or 14% decrease) while the proportion of people consuming poultry increased from 29.0% to 37.7% (or 30% increase) and fish/seafood from 15.6% to 21.4% (or 37% increase). Within the red meat category, declines were most pronounced for beef and lamb. Within the fish/seafood category, increases were most pronounced for canned fish. These changes were reflected in each SEIFA quintile, with the exception of the proportion consuming processed meat in the lowest SEIFA category which remained unchanged from 1995 to 2011–2012. The changes in meat types consumed were similar for both children and adults, although the proportion of children consuming processed meat was unchanged from 1995 to 2011–2012. 

### 3.2. Per-Capita Consumption

**Figure 1:**
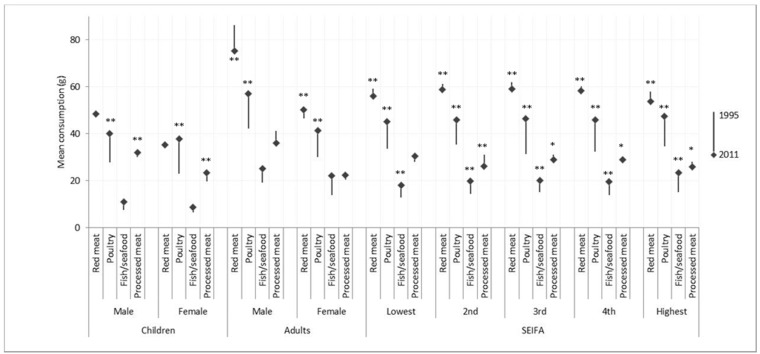
Per-capita mean consumption (g) of meat/poultry/fish from 1995 to 2011–2012, in children and adults, separated by gender, and SEIFA category. * *p*-value < 0.05 from Mann–Whitney U test; ** *p*-value < 0.01 from Mann–Whitney U test; ―◆ The start of the arrow indicates the per-capita consumption of meat/poultry/fish at 1995; the end of the arrow (capped by a square) indicates the per-capita consumption of meat/poultry/fish at 2011; the length of the arrow indicates the change of the per-capita consumption of meat/poultry/fish from 1995 to 2011–2012.

Per-capita consumption of total meat/poultry/fish increased from 1995 to 2011–2012 by 10.9% (137.2 g to 152.0 g, *p* < 0.001). The largest increases were seen for poultry (12.7 g difference) and fish/seafood (6.0 g difference) while smaller decreases in the consumption of red meat (−2.9 g difference) and processed meat (−0.8 g difference) were noted (Table 2). These trends differed somewhat between children and adults (Figure 1). For children, red meat and fish/seafood consumption remained unchanged, whereas processed meat consumption increased slightly (by 2.4 g) over this time period. For adults, no change in processed meat or fish/seafood consumption was noted, while red meat consumption decreased for males (by −10.9 g), but increased for females (by 3.6 g). 

Meat/poultry/fish consumption by SEIFA quintiles reflected the overall changes of increased per-capita poultry and fish/seafood intake and reduced intake of red meat and processed meat from 1995 to 2011–2012 in each quintile. The exception was in the lower SEIFA quintile, where no reduction in processed meat consumption was observed. More detailed per-capita data for all types of meat/poultry/fish are available in Supplementary Materials Table S3.

**Figure 2:**
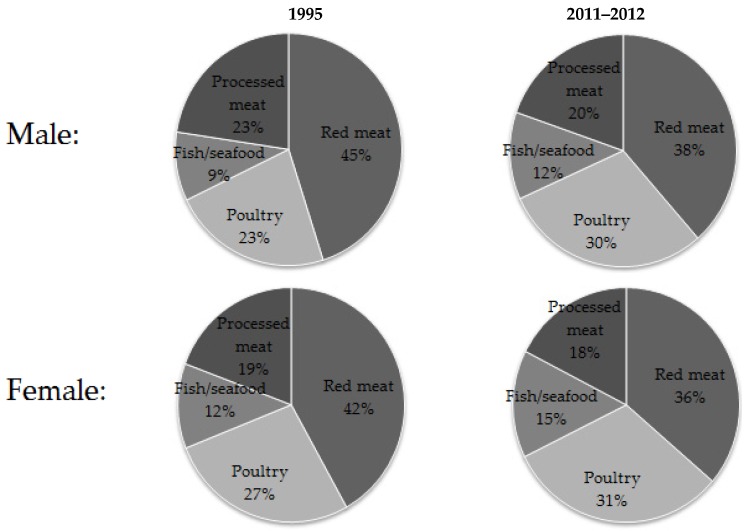
Per cent intake of different categories of meat in Australia, 1995 and 2011–2012.

From 1995 to 2011–2012, red meat remained the largest contributor of total meat/poultry/fish, followed by poultry (Figure 2). Compared with 1995, the population consumed a higher proportion of poultry and fish/seafood in 2011–2012 and a lower proportion of red meat and processed meat. 

### 3.3. Per-Consumer Consumption

Consumers of meat/poultry/fish had higher intakes in 2011–2012 compared with 1995 (149.5 g in 1995 versus 166.5 g in 2011–2012) with intakes of all meat/poultry/fish categories being significantly higher, with the exception of processed meat (Table 2). Per-consumer intakes of red meat and fish/seafood were at least 10 g higher in 2011–2012 compared with 1995 in all sub-groups examined (Supplementary Materials Table S4).

## 4. Discussion

Monitoring population dietary intakes is important to identify trends in food and nutrient intakes. In this study, we examined temporal changes in meat/poultry/fish consumption using disaggregated food intake data from 1995 NNS and 2011–2012 NNPAS. Over 2000 recipes were separated into individual ingredients to capture detailed data on amounts and types of meat consumed. Between 1995 and 2011–2012, the overall proportion of people consuming meat/poultry/fish remained relatively stable (at 92%) but a change in meat types consumed was observed. In 2011–2012, more people reported consuming poultry, mostly as chicken, and fewer reported consuming red meat such as beef and lamb. The per-capita consumption, which incorporates both proportion consuming and the amounts consumed, showed a small increase in the overall consumption of meat/poultry/fish. In all categories of meat/poultry/fish, the magnitude of change of consumption varied between children and adults, and by gender.

The strength of this study included the use of two national surveys, which are valuable datasets for monitoring meat/poultry/fish consumption on a population level in Australia. In both datasets, all composite dishes containing meat/poultry/fish were disaggregated into primary ingredients to be able to compare datasets. A matching database was available to match the meat types between surveys. However, there were several differences in the dietary assessment methodologies and food composition databases between the two surveys that limit comparability. Firstly, more detailed information was collected in the 2011–2012 NNPAS, including the preparation of meat cuts (e.g., diced, stir-fry strips were added), trimming practices (semi-trimmed was added to fully trimmed and untrimmed meats), and a larger selection of type of cooking oils used in meat preparation was available. Secondly, there was inconsistency in meat portion size captured between the two surveys, for example, the different sizes of small, medium, and large steaks recorded by the two surveys, which could introduce systematic error. For the 2011–2012 NNPAS, the measures data for meats was extensively updated based on products and food supply available at that time. However, there was limited information about the principles used to develop measures for the 1995 NNS. This limits comparability in portion size estimates. Thirdly, as highlighted by the ABS, there appears to be an increase in the level of under-reporting for both males and females between 1995 and 2011–2012, with males under-reporting to a greater extent in 2011–2012 compared with 1995 [7,8]. The impact of under-reporting to the assessment of meat consumption is unknown. Further work into the impact of under-reporting on the change in consumption patterns of different foods from the survey results is under investigation. These limitations mean that all results must be interpreted with caution, in particular the amounts or portions consumed. It is likely that the data on “proportion consuming” may be more reliable than the per-consumer and per-capita data. 

Despite the above limitations, the change in consumption of meat categories is likely to be a true reflection of current consumption patterns, as indicated by market share data with consumption of beef and lamb declining and chicken consumption increasing [5,6,7]. In our analysis, this finding was best reflected in the proportion of people consuming individual meat types on the day of the survey. In 1995, 56% of survey respondents consumed red meat and 29% poultry, compared with 49% and 38%, respectively, in 2011–2012. As the reported amounts consumed (per-consumer data) were higher in 2011–2012 for red meat than 1995, this translated into similar per-capita intakes (59 vs. 58 g) at both time points. Similar trends in red meat and chicken consumption have been observed in the United States using the NHANES survey from 1999 to 2007, where red meat consumption dropped from 105 to 85 g/day, while poultry consumption doubled from 25 to 55 g/day [2]. Evidence from a British birth cohort indicated that from 1999 to 2011–2012 the percentage of consumers of red meat and processed meat decreased whereas the percentage of consumers of white meat increased [11]. This change in consumption patterns is likely shaped by complex environmental factors, including pricing, availability, eating away from home, and food and lifestyle messages [2,11]. For example, chicken has become more affordable over the past five decades in Australia due to the significant improvements in efficiency and productivity in poultry related industries [5]. 

Epidemiological evidence suggests that disease risks vary by different choices of meat/poultry/fish consumption. Consumption of processed meat has been associated with an increased risk of coronary heart disease, diabetes, and colorectal cancer [12,13]. Unprocessed red meat has not been associated with risk of coronary heart disease or diabetes, although prolonged consumption of large amounts of red meat has been linked to risk of colorectal cancer [12,13,14,15,16]. No associations have been found for disease risk with increased consumption of poultry. Regular consumption of fish may be associated with reduced risk of heart disease, stroke, and dementia likely due to their long-chain omega-3 polyunsaturated fatty acids [17,18]. Current Australian dietary guidelines include advice on the amount of red meat and fish that are likely to be consistent with optimal health outcomes; the Guidelines also advise restrictions on the amount of “discretionary” foods, such as processed meat, that could be consumed. The Australian dietary recommendations for red meat intake are 32.5 g for children younger than nine years old and 65 g for all other age groups per day [1]. Findings from this analysis indicate that adult males reduced their consumption of red meat from an average of 86 to 75 g/day from 1995 to 2011–2012 while females consumed slightly more (from 46 to 50 g/day). There was, however, a reduction in the proportion of female consumers from 52% to 45%. As red meat is one of the most important food sources of easily absorbable iron [19], this may have consequences for the iron status of this vulnerable subgroup. 

The consumption of processed meat such as ham, bacon and sausages was reported by fewer people over time (from 44% in 1995 to 38% in 2011–2012) although per-capita intake remained relatively stable. Additionally, we observed a slight decline in per-capita consumption of processed meat in all socio-economic categories except for the lowest quintile. Evidence suggests that consumption of processed meat is associated with the risk of colorectal cancer and other chronic diseases [20]. One possible explanation for the slight reduction could be the increased awareness of the association between processed meat consumption and health outcomes. In addition, the NNPAS, as with all representative dietary surveys, is subject to under-reporting. The tendency for respondents to misreport the consumption of socially undesirable food choices has been identified in the survey by the ABS. The dietary guidelines of many countries recommend limiting or avoiding the consumption of processed meats. 

Fish and seafood consumption increased by 45% per-capita over the 16-year period, with the proportion of people reporting consuming canned fish nearly doubling over this time. However, fish and seafood remains the lowest reported category. These data are consistent with data collected by the Fisheries Research and Development Corporation [21] showing increases in consumption between 1991 and 1999, and from 2000 to 2013 as reported by the Australian Bureau of Agricultural and Resource Economics and Sciences (ABARES) [5]. 

## 5. Conclusions

Despite limitations in the comparison of consumption data over time, there has been a clear shift in the choice of poultry over red meat. Monitoring the trends and patterns of meat consumption is particularly pertinent to policy makers, researchers and other health professionals for the formulation of dietary recommendations and for estimating potential health outcomes.

## Figures and Tables

**Table 1:** Proportion (%) of people consuming meat/poultry/fish from 1995 to 2011–2012.

Proportion *	**Overall**			**Children**						**Adults**					
	**Overall**			**Male**			**Female**			**Male**			**Female**		
	**1995**	**2011–2012**	***p***	**1995**	**2011–2012**	***p***	**1995**	**2011–2012**	***p***	**1995**	**2011–2012**	***p***	**1995**	**2011–2012**	***p***
**Red meat**	56.1	48.6	<0.001	54.5	47.4	<0.001	51.9	44.6	<0.001	62.4	54.1	<0.001	52.0	45.4	<0.001
Beef	42.9	38.0	<0.001	44.5	39.9	0.01	41.4	36.6	0.004	48.3	42.4	<0.001	38.0	34.2	<0.001
Lamb	11.7	8.1	<0.001	8.7	6.6	0.02	8.8	5.6	0.001	12.8	9.3	<0.001	12.2	8.2	<0.001
Pork	7.5	7.5	0.93	5.7	5.4	0.74	6.0	5.9	0.78	8.5	8.9	0.69	7.4	7.3	0.73
Kangaroo	0.1	0.3	0.06	0.3	0.4	0.10	0.1	0.4	0.08	0.2	0.4	0.07	0.1	0.3	0.004
Game meat	0.1	0.1	0.47	0.2	0.0	0.06	0.0	0.1	0.22	0.1	0.2	0.77	0.1	0.1	0.38
**Poultry**	29.0	37.7	<0.001	27.5	37.5	<0.001	26.6	39.4	<0.001	29.8	37.8	<0.001	29.2	37.2	<0.001
Chicken	27.6	36.8	<0.001	26.7	37.1	<0.001	26.2	38.9	<0.001	28.2	36.6	<0.001	27.7	36.4	<0.001
Other poultry	1.9	1.3	0.61	0.8	0.6	0.05	0.6	0.9	0.08	2.1	1.6	0.07	2.3	1.2	<0.001
**Fish/seafood**	15.6	21.4	<0.001	9.6	13.7	0.001	11.8	14.3	0.03	17.4	22.5	<0.001	16.5	24.4	<0.001
Finfish	9.0	9.7	<0.001	6.1	6.9	0.97	8.0	7.3	0.39	10.1	11.0	0.13	9.1	10.0	0.31
Seafood	4.0	5.4	<0.001	2.3	3.0	0.05	2.2	3.2	0.07	4.6	5.8	0.006	4.2	6.3	<0.001
Canned fish	4.4	7.8	<0.001	1.7	4.2	<0.001	2.6	5.0	0.001	4.8	8.0	<0.001	5.2	9.5	<0.001
Fish/seafood products	0.8	1.6	<0.001	0.4	0.5	0.13	0.4	0.9	0.10	0.8	1.6	0.33	0.7	2.0	<0.001
**Organ/offal meat**	0.6	0.1	<0.001	0.1	0.1	0.06	0.1	0.0	0.57	0.8	0.2	<0.001	0.6	0.1	<0.001
**Processed meat**	44.0	37.8	<0.001	47.0	44.7	0.06	40.8	41.1	0.61	50.5	40.4	<0.001	38.4	32.8	<0.001
**Total meat/poultry/fish**	**91.7**	**91.3**	**0.16**	**90.6**	**90.8**	**0.09**	**90.1**	**90.0**	**0.11**	**94.6**	**93.2**	**0.16**	**89.9**	**90.1**	0.84
**Proportion**	**SEIFA**														
	**Lowest**			**2nd Quintile**			**3rd Quintile**			**4th Quintile**			**Highest**		
	**1995**	**2011–2012**	***p***	**1995**	**2011–2012**	***p***	**1995**	**2011–2012**	***p***	**1995**	**2011–2012**	***p***	**1995**	**2011–2012**	***p***
**Red meat**	55.8	48.0	<0.001	56.1	50.1	<0.001	55.6	48.8	<0.001	57.2	49.2	<0.001	55.5	47.2	<0.001
Beef	42.3	37.1	<0.001	41.8	39.2	0.06	43.4	39.5	0.004	44.2	37.5	<0.001	42.6	36.9	<0.001
Lamb	11.4	7.5	<0.001	13.0	8.2	<0.001	11.4	7.0	<0.001	11.7	8.9	0.001	10.8	8.8	0.01
Pork	6.9	8.3	0.07	8.0	7.2	0.28	7.5	7.6	0.81	7.8	7.6	0.77	7.1	6.8	0.58
Kangaroo	0.2	0.4	0.11	0.0	0.2	0.05	0.3	0.3	0.82	0.1	0.4	0.05	0.1	0.4	0.02
Game meat	0.1	0.3	0.25	0.2	0.1	0.32	0.1	0.1	0.14	0.0	0.0	0.58	0.0	0.0	0.45
**Poultry**	27.8	36.8	<0.001	28.6	36.0	<0.001	27.8	37.8	<0.001	28.8	37.3	<0.001	31.3	40.0	<0.001
Chicken	27.0	36.1	<0.001	26.9	35.4	<0.001	26.8	36.9	<0.001	27.5	36.4	<0.001	29.5	38.9	<0.001
Other poultry	1.2	0.8	0.19	2.3	1.0	<0.001	1.4	1.2	0.52	1.9	1.4	0.07	2.4	1.7	<0.001
**Fish/seafood**	13.9	17.8	<0.001	15.0	19.8	<0.001	16.0	19.6	<0.001	16.0	20.0	<0.001	16.6	23.6	<0.001
Finfish	8.3	9.0	0.39	8.9	9.9	0.18	9.1	9.5	0.62	9.8	9.0	0.32	9.0	10.6	0.04
Seafood	3.5	4.6	0.05	4.1	4.5	0.48	4.2	5.1	0.12	3.9	5.9	0.001	4.2	6.7	<0.001
Canned fish	3.7	6.1	<0.001	3.9	7.3	<0.001	4.1	7.9	<0.001	4.5	8.1	<0.001	5.6	9.5	<0.001
Fish/seafood products	0.6	0.7	0.585	0.9	1.8	0.08	0.9	0.9	0.10	0.4	0.9	0.97	1.0	1.2	0.47
Organ/offal meat	0.6	0.1	0.01	0.7	0.0	<0.001	0.4	0.2	0.33	0.7	0.1	0.002	0.5	0.1	<0.001
**Processed meat**	41.8	39.8	0.11	43.3	35.6	<0.001	44.6	40.5	0.003	45.1	39.2	<0.001	44.9	39.5	<0.001
**Total meat/poultry/fish**	**91.2**	**91.0**	**0.35**	**91.7**	**90.3**	**0.26**	**91.6**	**91.8**	**0.15**	**91.4**	**91.4**	**0.58**	**92.7**	**91.8**	0.14

* *p*-values for Chi-squared analysis between time points.

**Table 2:** Per-capita and per-consumer consumption (g) of meat/poultry/fish from 1995 to 2011–2012.

	Per-Capita Consumption (g)					Per-Consumer Consumption (g)				
	1995	2011–2012	Difference (g)	Difference (%)	*p* *	1995	2011–2012	Difference (g)	Difference (%)	*p* ^
	Mean (SD)	Mean (SD)				Median (25–75th Quartile)	Median (25–75th Quartile)			
**Red meat**	59.9 (89.0)	57.0 (88.4)	−2.9	−4.8	<0.001	81.0 (45.3–138.2)	98.1 (47.5–163.5)	17.1	21.2	<0.001
Beef	41.4 (75.2)	40.0 (75.8)	−1.4	−3.4	<0.001	70.9 (38.9–129.6)	83.3 (36.3–155.3)	12.4	17.5	<0.001
Lamb	11.4 (44.2)	9.6 (40.2)	−1.9	−15.8	<0.001	72.6 (43.5–126.5)	104.0 (61.0–156.0)	31.4	43.3	<0.001
Pork	6.8 (32.7)	7.0 (33.7)	0.2	2.9	0.35	69.1 (34.6–126.5)	75.0 (30.5–120.0)	5.9	8.5	0.54
**Poultry**	33.5 (73.7)	46.2 (86.7)	12.7	37.9	<0.001	90.0 (48.9–152.7)	95.0 (57.0–166.0)	5.0	5.6	0.001
Chicken	32.1 (72.2)	44.7 (84.8)	12.6	39.3	<0.001	92.0 (49.3–153.4)	93.6 (57.0–162.7)	1.6	1.7	0.021
Other poultry	1.4 (15.4)	1.5 (19.4)	0.1	7.1	<0.001	47.7 (15.0–101.8)	92.3 (33.6–151.9)	44.6	93.5	<0.001
**Fish/seafood**	15.2 (49.2)	22.1 (60.5)	6.9	45.4	<0.001	64.0 (35.5–117.5)	81.9 (46.1–124.0)	17.7	27.7	<0.001
Finfish	8.2 (38.3)	10.9 (43.1)	2.8	32.9	0.02	63.0 (34.0–117.5)	103.5 (53.7–136.8)	40.5	64.3	<0.001
Seafood	3.3 (23.0)	3.5 (21.2)	0.2	9.6	<0.001	60.8 (26.8–118.5)	46.6 (20.0–89.6)	−14.1	23.4	<0.001
Canned fish	2.8 (18.2)	5.9 (25.0)	3.1	110.7	<0.001	50.2 (30.0–84.3)	71.3 (40.0–95.0)	21.1	42.0	<0.001
**Processed meat**	29.1 (57.5)	26.5 (58.0)	−2.6	−8.9	<0.001	42.7 (21.1–84.0)	44.2 (18.8–93.5)	2.3	5.4	0.48
**Total**	**137.2 (124.7)**	**152.0 (128.9)**	**14.8**	**10.8**	**<0.001**	**118.1 (65.4–197.6)**	**139.1 (80.0–219.2)**	**21.1**	**17.8**	**<0.001**

* Independent *t*-test between time points; ^ *p*-value from Mann–Whitney U test. SD: Standard deviation; Meat/poultry/fish types reported by <1.0% of the population not reported in the table. See Figure 1 for subgroup information by age and gender; SD: Standard deviation.

## Supplementary Materials

The following are available online at http://www.mdpi.com/2072-6643/8/11/753/s1, Table S1: Characteristics of the respondents from the National Nutrition Survey 1995 (NNS 1995) and the Australian National Nutrition and Physical Activity Survey 2011–2012 (NNPAS 2011–2012), Table S2: Categorization of meat/poultry/fish, Table S3: Per-capita mean consumption (g) of meat/poultry/fish from 1995 to 2011–2012, Table S4: Per-consumer consumption (g) of meat/poultry/fish from 1995 to 2011–2012.
