# Supplementary material for: Changes in Meat/Poultry/Fish Consumption in Australia: From 1995 to 2011–2012
Source: Nutrients. 2016 Nov 24;8(12):753. doi: 10.3390/nu8120753 (PMC5188408; doi:10.3390/nu8120753)
Supplement: Supplementary file 1 [file nutrients-08-00753-s001.docx]

Supplementary Materials: Changes in Meat/Poultry/Fish Consumption in Australia: From 1995 to 2011–2012

Zhixian Sui, David Raubenheimer, Judy Cunningham and Anna Rangan

**Table S1.** Characteristics of the respondents from the National Nutrition Survey 1995 (NNS 1995) and the Australian National Nutrition and Physical Activity Survey 2011–2012 (NNPAS 2011–2012) *.

| **Characteristics** |  | **NNS 1995** | **NNPAS 2011–2012** |
| --- | --- | --- | --- |
| ***N*** |  | **13,858** | **12,153** |
|  |  | **Percentage % (*n*)** | **Percentage % (*n*)** |
| Gender | Male | 47.7 (6615) | 46.9 (5702) |
|  | Female | 52.5 (7273) | 53.1 (6451) |
| Age | 2–18 | 21.7 (3007) | 23.1(2812) |
|  | 19+ | 78.3 (10,851) | 76.9 (9341) |
| Socio-economic index for areas (SEIFA) | Lowest 20% | 18.1 (2512) | 18.4 (2238) |
|  | Second quintile | 18.7 (2587) | 20.3 (2464) |
|  | Third quintile | 19.1 (2650) | 20.0 (2432) |
|  | Fourth quintile | 21.3 (2945) | 17.9 (2177) |
|  | Highest 20% | 22.6 (3132) | 23.4 (2842) |
| Country of birth | Australia | 78.8 (10,925) | 75.3 (9157) |
|  | English speaking countries | 10.3 (1425) | 10.5 (1275) |
|  | Other | 10.9 (1508) | 14.2 (1721) |
| Level of Education | Postgraduate Degree/Graduate Diploma/Graduate Certificate | - | 6.3 (770) |
|  | Bachelor Degree | - | 13.3 (1615) |
|  | Advanced Diploma/Diploma | - | 8.1 (989) |
|  | Certificate | - | 18.6 (2263) |
|  | No non-school qualification | - | 34.5 (4190) |
|  | Not determined | - | 1.2 (146) |
| Employment status | Not applicable | 14.3 (1945) | 17.9 (2180) |
|  | Employed | 47.6 (6594) | 51.5 (6257) |
|  | Unemployed | 3.2 (445) | 2.2 (272) |
|  | Not in labour force | 14.9(2060) | 28.3 (3443) |
| Remoteness of location | Major cities of Australia | 50.3 (6968) | 64.1 (7788) |
|  | Inner regional Australia | - | 19.6 (2376) |
|  | Other | 20.2 (2805) | 16.4 (1989) |
|  | Rural areas | 29.5 (4085) | - |

***** Data owned by Australian Bureau of Statistics.

**Table S2.** Categorization of meat/poultry/fish.

| **Category** | **Type** |
| --- | --- |
| Red meat | Beef (including veal) |
|  | Lamb (including mutton) |
|  | Pork |
|  | Kangaroo |
|  | Game meats (including goat, rabbit, buffalo, camel, and venison) |
| Poultry | Chicken |
|  | Other poultry (including duck, turkey, quail, emu, ostrich, and pigeon) |
| Organ/offal meat | Liver |
|  | Kidney |
|  | Heart |
|  | Tongue |
|  | Tripe |
|  | Brain |
| Fish/seafood | Finfish (e.g., barramundi and salmon) |
|  | Seafood (e.g., prawn, oyster, and squid) |
|  | Canned fish (e.g., canned salmon and canned sardine) |
|  | Fish/seafood products (e.g., seafood stick, fish cake, seafood paste) |
| Processed meat | Processed/fresh sausage |
|  | Ham |
|  | Bacon |
|  | Nuggets |
|  | Salami (including other fermented meats) |
|  | Luncheon mammalian meat (e.g., devon and corned beef) |
|  | Processed luncheon poultry meat (e.g., smoked turkey) |
|  | Other processed meat (e.g., spam and beef jerky) |

**Table S3.** Per-capita consumption (g) of meat/poultry/fish from 1995 to 2011–2012.

|  |  |  | **1995** | | **2011–2012** | | **Difference** | ***p*-Value** |
| --- | --- | --- | --- | --- | --- | --- | --- | --- |
|  |  |  | **Mean** | **SD** | **Mean** | **SD** |  |  |
| Children  (2–18 years) | Male | Red meat | 47.04 | 71.10 | 48.39 | 79.09 | 1.35 | 0.664 |
|  |  | Poultry | 27.90 | 63.20 | 40.04 | 72.17 | 12.14 | 0.005 |
|  |  | Fish/seafood | 7.45 | 38.38 | 10.86 | 36.43 | 3.41 | 0.275 |
|  |  | Processed meat | 30.00 | 55.41 | 32.00 | 60.13 | 2.00 | 0.007 |
|  |  | Total meat/poultry/fish | 112.6 | 54.9 | 131.0 | 61.4 | 18.4 | 0.000 |
|  | Female | Red meat | 35.87 | 54.42 | 35.31 | 56.92 | −0.55 | 1.000 |
|  |  | Poultry | 22.98 | 55.90 | 37.76 | 67.68 | 14.77 | 0.001 |
|  |  | Fish/seafood | 6.46 | 24.11 | 8.78 | 27.39 | 2.32 | 0.796 |
|  |  | Processed meat | 19.70 | 36.51 | 23.34 | 46.55 | 3.64 | 0.003 |
|  |  | Total meat/poultry/fish | 85.0 | 40.1 | 105.2 | 44.7 | 20.2 | 0.000 |
| Adults  (19+ years) | Male | Red meat | 86.15 | 113.73 | 75.21 | 105.25 | −10.94 | 0.002 |
|  |  | Poultry | 42.22 | 89.90 | 56.90 | 107.31 | 14.67 | 0.002 |
|  |  | Fish/seafood | 19.07 | 60.04 | 25.12 | 63.81 | 6.05 | 0.298 |
|  |  | Processed meat | 41.18 | 73.41 | 35.99 | 76.47 | −5.20 | 0.313 |
|  |  | Total meat/poultry/fish | 189.1 | 75.6 | 193.4 | 76.9 | 4.3 | 0.000 |
|  | Female | Red meat | 46.45 | 67.61 | 50.06 | 79.08 | 3.61 | 0.005 |
|  |  | Poultry | 29.97 | 62.93 | 41.21 | 73.81 | 11.24 | 0.000 |
|  |  | Fish/seafood | 13.95 | 45.26 | 22.11 | 56.34 | 8.16 | 0.366 |
|  |  | Processed meat | 20.52 | 42.45 | 22.23 | 53.22 | 1.71 | 0.967 |
|  |  | Total meat/poultry/fish | 111.3 | 47.1 | 135.7 | 55.3 | 24.4 | 0.000 |
| SEIFA quintiles  (2+ years) | 1st | Red meat | 59.05 | 91.50 | 56.06 | 88.70 | −3.00 | 0.003 |
|  |  | Poultry | 33.57 | 76.08 | 45.10 | 83.84 | 11.53 | 0.000 |
|  |  | Fish/seafood | 12.81 | 46.15 | 18.17 | 51.41 | 5.37 | 0.000 |
|  |  | Processed meat | 28.03 | 54.33 | 30.44 | 64.64 | 2.41 | 0.162 |
|  |  | Total meat/poultry/fish | 133.6 | 63.8 | 149.9 | 64.2 | 16.3 | 0.000 |
|  | 2nd | Red meat | 61.13 | 90.50 | 58.84 | 87.53 | −2.29 | 0.001 |
|  |  | Poultry | 35.34 | 81.45 | 45.82 | 90.95 | 10.48 | 0.000 |
|  |  | Fish/seafood | 14.36 | 51.98 | 19.78 | 52.85 | 5.42 | 0.000 |
|  |  | Processed meat | 31.11 | 62.72 | 26.27 | 64.96 | −4.85 | 0.007 |
|  |  | Total meat/poultry/fish | 142.3 | 68.4 | 150.8 | 66.8 | 8.5 | 0.000 |
|  | 3rd | Red meat | 62.02 | 95.45 | 58.90 | 90.53 | −3.12 | 0.003 |
|  |  | Poultry | 31.33 | 68.37 | 46.46 | 83.13 | 15.14 | 0.000 |
|  |  | Fish/seafood | 15.21 | 49.54 | 20.13 | 57.50 | 4.93 | 0.001 |
|  |  | Processed meat | 31.00 | 61.21 | 29.00 | 61.87 | −2.00 | 0.010 |
|  |  | Total meat/poultry/fish | 139.8 | 64.6 | 155.1 | 65.8 | 15.3 | 0.000 |
|  | 4th | Red meat | 60.10 | 86.62 | 58.16 | 90.96 | −1.94 | 0.009 |
|  |  | Poultry | 32.45 | 69.42 | 45.91 | 89.38 | 13.46 | 0.000 |
|  |  | Fish/seafood | 13.97 | 46.95 | 19.52 | 53.95 | 5.55 | 0.000 |
|  |  | Processed meat | 28.00 | 52.99 | 29.00 | 62.87 | 1.00 | 0.028 |
|  |  | Total meat/poultry/fish | 134.8 | 60.0 | 152.8 | 67.9 | 18.0 | 0.000 |
|  | 5th | Red meat | 57.78 | 82.18 | 53.79 | 84.75 | −3.99 | 0.005 |
|  |  | Poultry | 34.68 | 73.32 | 47.41 | 86.18 | 12.73 | 0.000 |
|  |  | Fish/seafood | 15.08 | 50.89 | 23.29 | 58.25 | 8.21 | 0.000 |
|  |  | Processed meat | 28.00 | 56.28 | 26.00 | 60.27 | −2.00 | 0.020 |
|  |  | Total meat/poultry/fish | 135.9 | 61.2 | 151.3 | 64.1 | 15.4 | 0.000 |

**Table S4.** Per consumer consumption (g) of meat/poultry/fish from 1995 to 2011–2012.

| **Per Consumer Consumption** | | | | | | | | |
| --- | --- | --- | --- | --- | --- | --- | --- | --- |
|  |  |  | **1995** | | **2011–2012** | | **Difference** | ***p*-Value** |
|  |  |  | **Median** | **25–75th Quartile** | **Median** | **25–75th Quartile** |  |  |
| Children  (2–18 years) | Male | Red meat | 62.58 | 36.75–126.72 | 78.40 | 38.84–147 | 15.82 | 0.055 |
|  |  | Poultry | 76.18 | 47.66–138.92 | 81.35 | 50–138.7 | 5.17 | 0.498 |
|  |  | Fish/seafood | 47.93 | 30.72–107.2 | 67.59 | 33–117.8 | 19.66 | 0.068 |
|  |  | Processed meat | 46.55 | 20–89 | 45.43 | 19.69–89 | −1.12 | 0.644 |
|  |  | Total meat/poultry/fish | 104.0 | 57.68–177.72 | 114.0 | 64.18–192.15 | 10.0 | 0.544 |
|  | Female | Red meat | 53.10 | 30.55–100 | 64.00 | 29.21–115.92 | 10.90 | 0.155 |
|  |  | Poultry | 67.00 | 40–113.79 | 74.10 | 42.51–114 | 7.10 | 0.016 |
|  |  | Fish/seafood | 39.87 | 24–80 | 51.31 | 26–86.28 | 11.44 | 0.052 |
|  |  | Processed meat | 35.97 | 17–67.62 | 33.30 | 17–74.06 | −2.67 | 0.734 |
|  |  | Total meat/poultry/fish | 84.75 | 47.78–143 | 97.0 | 55.3–163.2 | 12.25 | 0.090 |
| Adults  (19+ years) | Male | Red meat | 108.00 | 60.48–184.97 | 119.40 | 59.92–189.69 | 11.40 | 0.046 |
|  |  | Poultry | 118.00 | 64.32–191.1 | 116.94 | 66–195.7 | −1.07 | 0.203 |
|  |  | Fish/seafood | 79.20 | 48–144 | 95.00 | 61.1–152.5 | 15.80 | 0.107 |
|  |  | Processed meat | 54.43 | 26.4–105.6 | 50.63 | 24.95–101.41 | −3.81 | 0.012 |
|  |  | Total meat/poultry/fish | 171.0 | 101.25–266.46 | 178.75 | 104–273.23 | 7.75 | 0.540 |
|  | Female | Red meat | 71.05 | 44.53–135.48 | 96.97 | 49–156 | 25.92 | 0.230 |
|  |  | Poultry | 84.67 | 49.22–143 | 89.25 | 55.8–149 | 4.58 | 0.812 |
|  |  | Fish/seafood | 60.50 | 37.95–113.05 | 79.80 | 46.08–121.6 | 19.30 | 0.036 |
|  |  | Processed meat | 34.37 | 18.33–73.44 | 40.00 | 17–76.88 | 5.63 | 0.721 |
|  |  | Total meat/poultry/fish | 114.0 | 66.52–180.9 | 129.25 | 78.15–198 | 15.25 | 0.057 |
| SEIFA quintiles  (2+ years) | 1st | Red meat | 76.34 | 43.41–150.69 | 98.00 | 45.25–164.34 | 21.66 | 0.000 |
|  |  | Poultry | 93.10 | 57.04–162 | 100.00 | 59.39–162.32 | 6.90 | 0.267 |
|  |  | Fish/seafood | 63.73 | 39.8–124.33 | 86.10 | 46–139.62 | 22.37 | 0.001 |
|  |  | Processed meat | 43.51 | 21.53–90.23 | 46.00 | 20.66–94 | 2.49 | 0.237 |
|  |  | Total meat/poultry/fish | 125.5 | 70–204.4 | 140.0 | 81.35–214.83 | 14.5 | 0.005 |
|  | 2nd | Red meat | 83.12 | 47.52–156 | 97.06 | 50–165 | 13.94 | 0.000 |
|  |  | Poultry | 89.25 | 53.41–163.02 | 96.00 | 58.25–166 | 6.75 | −1.377 |
|  |  | Fish/seafood | 67.00 | 40.99–123.36 | 80.64 | 46.23–124 | 13.64 | −2.734 |
|  |  | Processed meat | 47.00 | 21–92.18 | 45.00 | 17.4–91 | −2.00 | 0.362 |
|  |  | Total meat/poultry/fish | 130.73 | 72–209.48 | 142.49 | 80.84–216.34 | 11.76 | 0.000 |
|  | 3rd | Red meat | 83.08 | 46.37–157.65 | 100.74 | 47.39–167.58 | 17.66 | 0.001 |
|  |  | Poultry | 95.96 | 55.71–160.72 | 97.59 | 59.76–177.75 | 1.63 | 0.045 |
|  |  | Fish/seafood | 69.14 | 38–125 | 83.09 | 46.23–126.61 | 13.94 | 0.014 |
|  |  | Processed meat | 46.88 | 21.5–93.29 | 45.73 | 19–94 | −1.15 | 0.873 |
|  |  | Total meat/poultry/fish | 131.69 | 74.1–211.32 | 138.28 | 80.75–224.83 | 6.59 | 0.573 |
|  | 4th | Red meat | 79.22 | 46–147 | 99.30 | 49.07–156.49 | 20.07 | 0.000 |
|  |  | Poultry | 90.74 | 52.15–160 | 89.25 | 56–164.26 | −1.49 | 0.344 |
|  |  | Fish/seafood | 60.00 | 37.2–117.8 | 82.50 | 38.7–128 | 22.50 | 0.001 |
|  |  | Processed meat | 42.00 | 20.64–85.86 | 44.50 | 18.08–94 | 2.50 | 0.094 |
|  |  | Total meat/poultry/fish | 126.5 | 70–206.24 | 136.88 | 76–219.55 | 10.38 | 0.140 |
|  | 5th | Red meat | 81.53 | 45.27–147 | 95.54 | 45.08–156.49 | 14.01 | 0.003 |
|  |  | Poultry | 87.35 | 47.66–154.32 | 93.00 | 51.67–160.15 | 5.65 | 0.032 |
|  |  | Fish/seafood | 63.82 | 39.13–120.06 | 79.80 | 46.04–125 | 15.98 | 0.000 |
|  |  | Processed meat | 40.00 | 19.36–77.77 | 42.00 | 17.4–84 | 2.00 | 0.124 |
|  |  | Total meat/poultry/fish | 125.12 | 70.57–208 | 137.50 | 78.66–221.99 | 12.38 | 0.000 |
